# Transitions to crop residue burning have multiple antecedents in Eastern India

**DOI:** 10.1007/s13593-024-00983-3

**Published:** 2024-11-07

**Authors:** E. Urban Cordeiro, A. Samaddar, S. Munshi, A. Ajay, D. G. Rossiter, R. K. Sohane, R. Malik, P. Craufurd, P. Pingali, A.J. McDonald

**Affiliations:** 1 Soil & Crop Sciences, Cornell University, Ithaca, USA; 2 International Rice Research Institute (IRRI), New Delhi, India; 3 International Maize and Wheat Improvement Center (CIMMYT), Patna, India; 4 Bihar Agricultural University, Bhagalpur, India; 5 International Maize and Wheat Improvement Center (CIMMYT), Kathmandu, Nepal; 6 Tata-Cornell Institute for Agriculture and Nutrition, Cornell University, Ithaca, USA

**Keywords:** Crop burning, Air quality, Bihar, Indo-Gangetic Plain, Mixed crop-livestock systems, Mixed methods, Socio-technical drivers

## Abstract

Far removed from the agricultural fire “hotspots” of Northwestern India, rice residue burning is on the rise in Eastern India with implications for regional air quality and agricultural sustainability. The underlying drivers contributing to the increase in burning have been linked to the adoption of mechanized (combine) harvesting but, in general, are inadequately understood. We hypothesize that the adoption of burning as a management practice results from a set of socio-technical interactions rather than emerging from a single factor. Using a mixed methods approach, a household survey (*n* = 475) provided quantitative insights into landscape and farm-scale drivers of burning and was complemented by an in-depth qualitative survey (*n* = 36) to characterize decision processes and to verify causal inferences derived from the broader survey. For communities where the combine harvester is present, our results show that rice residue burning is not inevitable. The decision to burn appears to emerge from a cascading sequence of events, starting with the following: (1) decreasing household labor, *leading to* (2) decreasing household livestock holdings, *resulting in* (3) reduced demands for residue fodder, *incentivizing* (4) adoption of labor-efficient combine harvesting and subsequent burning of loose residues that are both difficult to collect and of lower feeding value than manually harvested straw. Local demand for crop residues for livestock feeding plays a central role mediating transitions to burning. Consequently, policy response options that only consider the role of the combine harvester are likely to be ineffective. Innovative strategies such as the creation of decentralized commercial models for dairy value chains may bolster local residue demand by addressing household-scale labor bottlenecks to maintaining livestock. Secondary issues, such as timely rice planting, merit consideration as part of holistic responses to “bend” agricultural burning trajectories in Eastern India towards more sustainable practices.

**Supplementary Information:**

The online version contains supplementary material available at 10.1007/s13593-024-00983-3.

## Introduction

### Residue burning in Eastern India

**Fig. 1:**
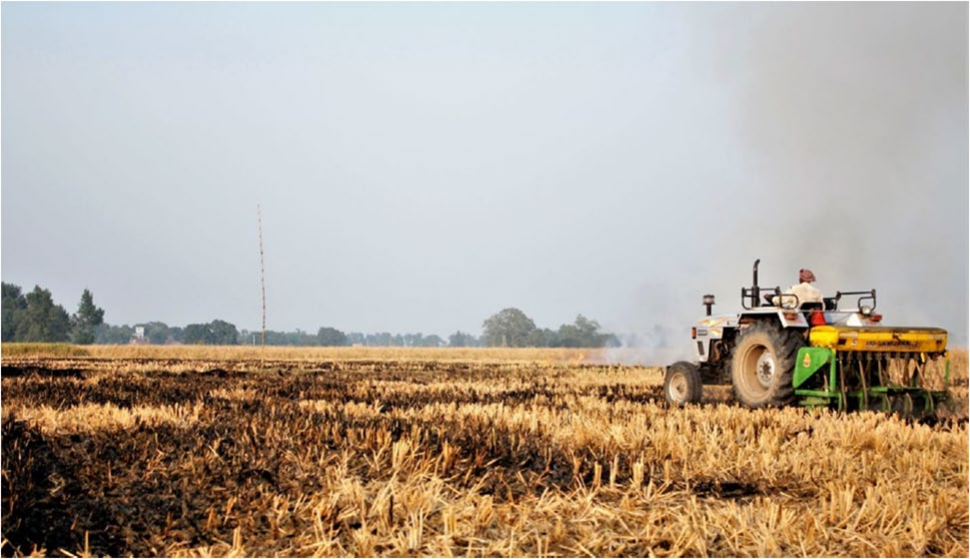
Buxar, Bihar, showing partially burnt rice residue with zero-till wheat sowing is in progress (PC: Anurag Ajay)

Rice residue burning (Figure 1) has risen by 142% over the past two decades in the densely populated state of Bihar in Eastern India (Urban Cordeiro et al. 2023). Geographically distanced from the burning “hotspot” in the states of Punjab and Haryana in Northwest India, this rapid rise—albeit from a low base—has received limited national attention but will likely exacerbate existing public health burdens caused by poor air quality. Patna, the capital city of Bihar, is already identified as one of the most air-polluted metropolitan regions in India (Nair et al. 2021). On a seasonal basis, crop residue burning is known to contribute to regional air pollution in South Asia and heightened levels of PM_2.5_ exposure (i.e., small particulate pollution that is most damaging to human health), increasing risks for a host of respiratory and cardiovascular diseases (Bikkina et al. 2019; Liu et al. 2018; Ravindra et al. 2019). In the predominant rice-wheat cropping system, the timing of rice residue burning in the fall coincides with the peak period of poor regional air quality (Bikkina et al. 2019). From a food security perspective, residue burning can also contribute to long-term productivity losses by affecting nutrient and carbon cycling in the soil (Shi et al. 2014).

**Fig. 2:**
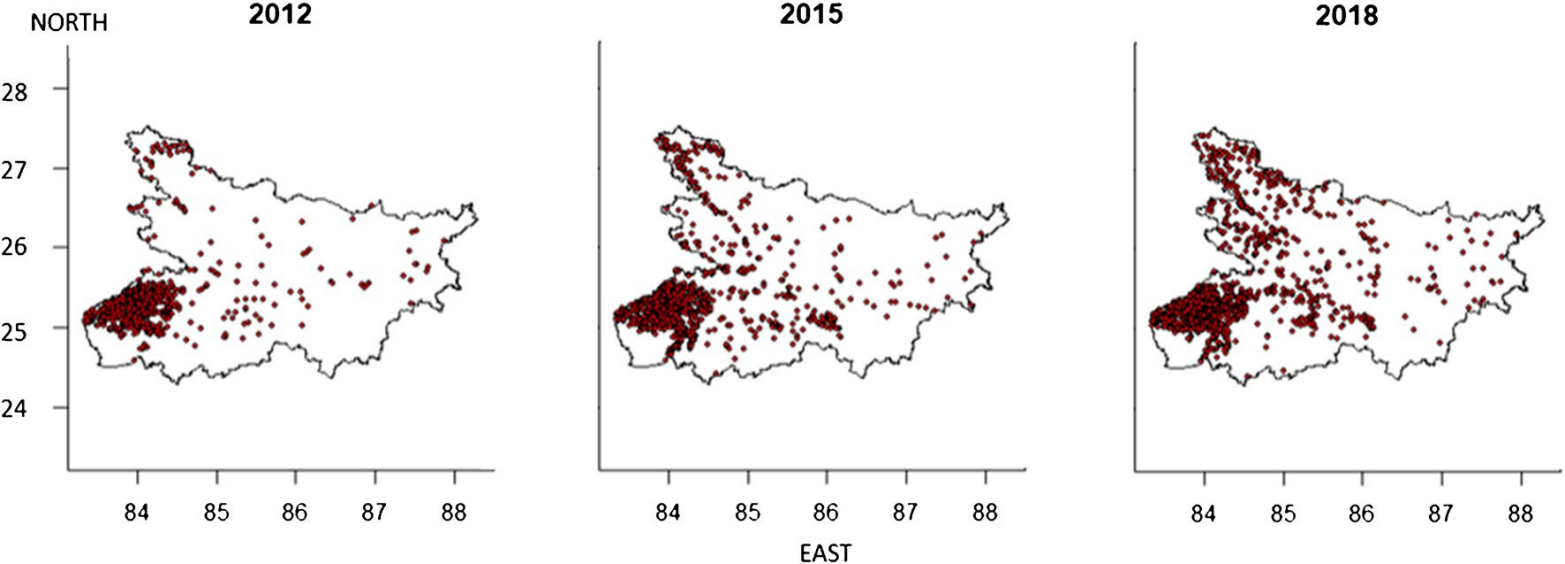
Visible Infrared Imaging Radiometer Suite (VIIRS) remotely sensed fire observations during rice harvest season in Bihar (Urban Cordeiro et al. 2023).

In Bihar, rice residue fires are both increasing in frequency over time, particularly in the southwest corner of the state, and expanding spatially, particularly eastward (Urban Cordeiro et al. 2023) (Figure 2). The drivers of these changes in Eastern India within the Indo-Gangetic Plains have not yet been studied, but experiences from Northwest India provide insights into possible causes. In Northwest India, rice residue burning has been positively associated with the adoption of the combine harvester, which breaks and bends the straw rendering it low quality for other purposes (Kumar et al. 2015); crop intensification, which increases the residue load that the subsequent wheat crop must be planted into (Gupta et al. 2004); a short planting time incentivizing quick residue removal (Balwinder-Singh et al. 2019a, b); and reduced labor availability and increased costs, which make manual removal of the residues economically unattractive (Lopes et al. 2020). However, Eastern India has ecological, social, cultural, and economical differences from the Northwest that may influence burning decisions and therefore merits separate consideration, particularly as it relates to crop-livestock relations at household and landscape scales.

The livestock systems in Eastern India play a central role in the fate of rice residues. Bihar is characterized by small landholdings and mixed farming systems with close functional integration between livestock and cropping systems. The dairy system is primarily composed of small herds at the household level, averaging two animals, but may be experiencing structural changes as the demand for milk increases, markets formalize, breed preference shift from indigenous to crossbreeds, and feeding practices change towards supplementation and other market-based approaches that draw feedstocks from beyond the household or village levels. From 2007 to 2012, the total number of cattle has decreased by 0.53% per year but with a reversal of this trend forecasted due to economic growth and dietary transitions in the state (Bihar Livestock Master Plan 2018). There is a stronger feeding preference for rice straw in Eastern India compared to Northwest India, where straw feeding is largely from wheat (Erenstein and Thorpe 2010). A study in the Terai region of Nepal found that combine harvester use, which is associated with burning, was 26% less likely to be adopted by households with livestock (Bajracharya et al. 2021).

Drivers of burning may also be related to the changing rural labor dynamics. Along with the state of Uttar Pradesh, Bihar, has the highest rate of out-migration in India. This generally occurs from rural to urban areas outside of the state but there also are a substantial number of internal migrants who remain in Bihar (Nayyar and Kim 2018). Climate hazards influence migration in India as well, causing an estimated 8% of rural-urban migration between 2005 and 2012 (Sedova and Kalkuhl 2020). While India’s National Rural Employment Guarantee Act (NREGA) (https://nrega.nic.in) was enacted in 2005 to provide rural work opportunities to reduce urban migration, Bihar was among the states that did not experience a slowdown in prior demographic transitions (Imbert and Papp 2020). As rural wages rise—often as a response from economic growth in sectors outside of agriculture—“labor-saving technologies,” such as the combine harvester, see substantial growth. Cropping systems with multiple cropping seasons in a single year, such as in rice-wheat systems, have additional labor challenges, as harvest and planting activities are needed within the same short window (Pingali 2007). Changes in labor availability and cost will inevitably incentivize further technological changes like mechanization and also, for some households, wholesale changes in agricultural systems (e.g., away from livestock).

Research responding to sustainability challenges in agriculture, such as crop residue burning, often first focus on proximal causes (i.e., mechanized harvesting) and narrow technological solutions (i.e., conservation agriculture) without considering underlying drivers of change and system dynamics beyond the scale of individual production fields. In the case of burning in Eastern India, we hypothesize that multiple social, economic, and biophysical factors are influencing transitions to burning and that effective policy interventions can capitalize on insights gained by studying these factors with an integrated systems analysis. We address this knowledge gap by further studying farmer decision-making processes, contextualized through the larger body of international literature. Farmer decision-making studies within the smallholder farmer context have largely been situated in a specific disciplinary domain, such as economics, anthropology, and psychology, often relying heavily on theoretical frameworks like profit-maximizing, theory of planned behavior and bounded rationality (Singh et al. 2016). However, recent efforts have embraced more integrative approaches, considering both behavioral factors and physical constraints (Malawska and Topping 2016). Moreover, researchers have begun exploring not only individual factors influencing decision-making (Hayden et al. 2021) but also the sequential nature of these decisions (Robert et al. 2016). Transitioning from the focus on proximal causes and narrow technological solutions in responding to sustainability challenges in agriculture, such as crop residue burning, we utilize a comprehensive characterization of landscapes and change processes in our study by using a mixed method approach.

### Characterizing landscapes and change processes

In this study, we combine quantitative and qualitative evidence to understand how various actors and interactions in agricultural landscapes function and change processes occur. Agricultural burning has proven very difficult to stop in Northwest India despite an array of “sticks” (e.g., fines for those that burn) and “carrots” (e.g., subsidies for “no burn” agricultural machinery like the Happy Seeder) provided by state governments (Shyamsundar et al. 2019). Based on the experience in the Northwest, there is an emerging consensus that effective strategies for burning prevention must consider the underlying driving factors that incentivize the practice as well as the policy and technological levers that may encourage residues to be valued as resources rather than waste products that should be burned (Downing et al. 2022).

In this paper, we leverage a mixed methods approach to contextualize the practice of burning in a broader systems context in order to inform targeted policy action. This study has two objectives: to (1) identify household and landscape characteristics that determine the fate of rice residues, particularly burning and (2) from these, characterize the structural changes and decision processes that lead towards rice residues being burned as a waste product. Our methodology involved initial focus groups (5 groups) to inform the survey design, followed by a quantitative survey (475 households). To capture nuanced interactions, we also conducted qualitative interviews (36 households).

## Materials and methods

### Quantitative methods and random forest analysis

#### Quantitative data collection

The quantitative data used for the analysis of this paper is a subset and refinement of two prior surveys. First, the Cereal Systems Initiative for South Asia (csisa.org) and the Indian Council of Agricultural Research’s Krishi Vigyan Kendra (KVK) deployed a crop diagnostics survey (2017 rice season; 7597 households) in 39 districts in Bihar and neighboring parts of Uttar Pradesh. In each district, 30 villages were selected, with seven households per village, resulting in 210 households per district (Ajay et al. 2022) (https://data.cimmyt.org/dataverse/csisadvn). Second, two crop residue burning surveys (460 households and 475 households) were conducted as sub-modules to the crop diagnostics survey and deployed by the authors in the Bihar districts where more than 100 of the 210 households were producing rice (30 districts). The second of these surveys (475 households) is used for this study. To guide the development of the initial crop residue burning survey, preliminary in-person focus group discussions (five focus groups, ranging from four to eight participants) took place over the course of a week in January 2020. The initial survey sampling frame included ten households per district. Six districts with high combine harvester adoption (Bhabua, Buxar, Bhojpur, Rohtas, Nawada, and Lakhisarai) were oversampled for a total of 20 households in those districts. For each district, enumerators were given a randomized list of 30 possible households. The enumerator called the first on the list and moved to the subsequent one if there was no response. This continued until 10 (or 20 for the oversampled districts) interviews were conducted. The survey was conducted in May 2020 via phone due to the ongoing COVID-19 pandemic.

The second crop residue burning survey (475 households), from which the primary quantitative data for this study is derived, was a further exploration of the initial crop residue burning survey to include additional questions related to livestock, milk markets, residue markets, and labor. Sampling targeted all previous households (235 households), additional first-time households (139 households), and purposefully selected households that previously reported harvesting their rice crop with a combine harvester in the crop diagnostics survey (101 households). During analysis, the data from this survey was linked to respondents’ prior crop diagnostics survey data to provide a comprehensive dataset.

#### Random forest analysis

Our data-driven approach used random forest analysis (Breiman 2001), as implemented by the “ranger” package (Wright and Ziegler 2017) of the R Environment for Statistical Computing (R Core Team 2022), to study which predictor variables are associated with the rice residue burning. A random forest model was selected over a standard statistical model, given that the relationship between variables in the dataset were assumed to be complex and non-linear. Additionally, the target variable, burning likelihood, was skewed towards zero. The algorithm combines numerous decision trees to build an ensemble of trees called a “forest” to arrive at a model of how the predictive factors affect an outcome. This can then be used to predict an outcome for any combination of predictors in the feature space used to build the random forest. It also reveals which predictive factors are most important in the predictive model, thus suggesting interpretations as to possible driving factors. The first step in random forest analysis is to identify the target variable, and then covariates which are suspected to have a predictive relation with the target variable.

Here, the target variable is the burn probability at each household location. We based this on previous burning history. However, because of punitive burning laws, very few respondents in our survey admitted to burning, yet we know, anecdotally, that many of our respondents have in fact burned residue in the past. This knowledge was gained through conversations with village leaders, neighbors, and households. To address this deficiency, we used remote-sensing surrogates, namely 2020 MODIS 2003) (1 km spatial resolution; October 1–December 31, 2020) and VIIRS Active Fire Products (Schroeder et al. 2014) (375 m spatial resolution; October 1–December 31, 2020) to create a “burn likelihood” raster for the 2020 cropping season. Imagery was masked using the 2016 ESA CCI Land Cover Product (Defourny et al. 2016)—merging the rainfed cropland, irrigated cropland, and mosaic cropland classes—to include only cropland areas. Only dates during the rice residue burning season were used (i.e., October 1 to December 31). To overcome errors of omission in the datasets, we used both MODIS and VIIRS observations, created a kernel density estimation map, and normalized the continuous surface to find a “burn likelihood” value for each grid cell. To begin, we combined the two remote sensing products by creating a 1-km buffer around the VIIRS points and removed MODIS points within the buffer to not double count, following similar methods used by Liu and colleagues (2020). Then, a kernel density map, with a bandwidth of 10 km, was created from the fire points. The kernel density is a continuous surface that expresses the density of points (in this case, fire occurrences) in a neighborhood of each point on the surface. This continuous surface was normalized by the maximum density across the map and then converted to a gridded map, with each cell being assigned a “burn likelihood” value on a scale of 0 to 1, with 0 being no burn likelihood and 1 being the highest likelihood of burning in the state. The “burn likelihood” value of each household surveyed was extracted by map overlay, using the GPS locations of the households.

With the target variable established, we then drew up a list of candidate predictor variables, considered to have a possible predictive relation with burning. These included landholding size, labor, livestock, mechanization, income source, and market connectivity (Table 1). Variables were derived from a variety of sources due to data availability, including those from the prior crop diagnostics survey, the crop residue burning survey, and satellite information. During the random forest analysis, variable importance and partial dependency plots were utilized to rank the predictors and to characterize the nature of the association between the most important predictors and rice residue burning, and then limit the model to the most parsimonious that still explained most of the variability in the target variable.

**Table 1:** Variables used in the random forest analysis. The target variable is Burn.likelihood. The sources are abbreviated accordingly: Crop Diagnostics Survey (CDS) and Residue Burning Survey (RBS). The Harvest.date variable was derived from MODIS 250 m EVI (Terra & Aqua) data at an 8-day interval and in temporal alignment with the remotely sensed burning products of the same year.

Variable	Description	Source
Burn.likelihood	Remotely sensed derived value of the likelihood of rice burning occurrence	Satellite
No.dairy.animals	Number of adult dairy animals owned by household (buffalo or cow)	RBS
Stocking.density	Number of adult dairy animals by cultivated land	RBS
Dairy.animal.trend	Number of household livestock present day compared to 5 years ago (Increase, decreased, or stayed the same)	RBS
Cultivated.land	Land under cultivation during rice season	CDS
HH.mem	Number of members in household (any sex or age)	CDS
HH.mem.ag	Number of household members involved in on-farm agriculture	CDS
No.HH.males	Number of male members in household (any age)	RBS
No.HH.females	Number of female members in household (any age)	RBS
Combine.percent	Percentage of rice crop the household harvested by combine	RBS
Rice.yield	Yield (quintal) per area	CDS
Rice.variety	Varietal type of rice grown	CDS
Market.distance	Distance to market (km)	CDS
Ag.income.share	Percentage of household income from on-farm agricultural activities	CDS
Residue.sourced	Whether the household sourced residue (purchased or free)	RBS
Residue.sold	Whether the household sold rice residue (farmer, middleman, etc.)	RBS
Harvest.date	Rice harvest date extracted from 2020 MODIS raster	Satellite
Market.share	Percentage of agricultural output sold	CDS

### Qualitative methods

#### Qualitative theoretical framework

**Fig. 3:**
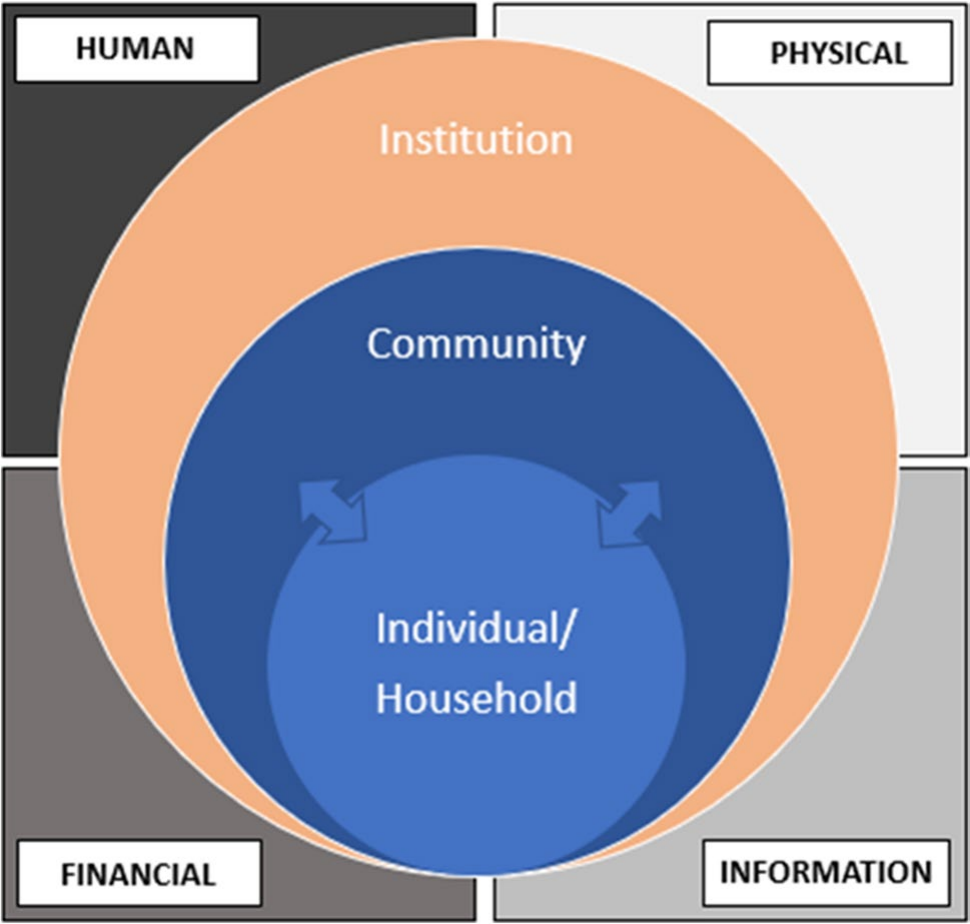
Livelihood Platforms Approach. Adapted from Brown et al. (2017).

The Livelihood Platforms Approach (LPA) (Brown et al. 2017) was used to examine farmer decision-making related to residue burning. Building off the sustainable livelihoods approach (Carney 1998), the framework has four resource categories, namely, physical, financial, human, and informational (Figure 3). The LPA’s hierarchical structure enables the evaluation of these resource categories at the individual, household, community, and institutional levels. The LPA is used in this study to identify the enabling environment for burning to identify possible entry points for prevention. In the results, the resource and level are indicated in parentheses, as appropriate, to align with the theoretical framework.

#### Qualitative data collection and analysis

Qualitative interviews (36 respondents from 18 households) were conducted in Buxar district, southwest Bihar. Both the male and female heads of household were interviewed, without the presence of their spouse. The selection of Buxar district was based on survey findings and local experts who supported our efforts to identify a location where the combine harvester was readily available, rice residue burning was present, and average livestock holding was similar to the state average. Three villages (Rajapur, Lalachak, and Chhotaki) were selected based on those characteristics. Through a criterion-based sampling method (Patton 2015), six households per village were selected. Three of these households had a landholding size below the district average and three over the average (1 ha). Three households had herd sizes above the district herd size average and three households were below the average (2 livestock) were targeted. From this, enumerators interviewed a roughly even split from each category of burn versus no-burn households (2–3 households burning in each category). The interviews were conducted in-person by two local, female interviewers during May–June 2022. These interviewers were trained and supervised by the authors for the duration of the data collection.

Interviews were conducted in either Hindi or Bhojpuri languages, depending on the respondent’s preference. Interviews were translated and transcribed in English by a third party and reviewed by the researchers and interviewers for clarity and corrections. The de-identified transcriptions were coded in Dedoose (www.dedoose.com) using a pre-developed codebook compiled by the researchers according to the LPA framework. Key themes were then synthesized through a code mapping exercise drawn and discussed by the researchers to determine key drivers of change in this ecology, the interactions among them, and the sequence of change.

## Results

### Quantitative results: livestock, combine harvester usage, and straw management

#### ***Descriptive statistics***

There were 475 total respondents, with 374 drawn from a random subset of the pre-existing crop diagnostics survey that was designed to be representative at the Bihar State level (Ajay et al. 2022) and 101 purposefully selected to increase the number of combine harvester user respondents. Descriptive statistics are derived from the representative sample surveys (*n* = 374) to avoid the geographic bias introduced by the purposive sample. The full dataset was used for the subsequent analysis that explores drivers of combine harvester use and agricultural burning. Additional results can be found in the Supplemental Information.

##### Livestock

Of the state-wide sample (*n* = 374), 330 (86.7%) households had livestock and 44 did not. In the last 5 years, 218 (61.2%) households reported a decrease in their total livestock numbers, 61 (17.1%) reported an increase, and 77 (21.6%) reported no change (Table 2). Reasons cited for a decrease in livestock included reduced household labor to care for the livestock (*n* = 101), selling due to needed cash (*n* = 75), low profitability (*n* = 59), reduced availability of hired labor (*n* = 33), and/or loss of milk market opportunities (*n*= 30). For the households that increased their livestock holdings (*n* = 61), a majority cited an increase in household labor (*n* = 27) and others an increase in milk profitability (*n* = 15), and/or an increase of household milk consumption (*n* = 14). Around 30% of all households reported wanting to change the type of livestock they keep, with 38 (35.8%) considering the transition from the indigenous cow to crossbred, 27 (25.5%) considering cow to buffalo, 20 (18.9%) considering buffalo to cow, 18 (17.0%) considering crossbred to indigenous cow. Reasons cited included higher milk quality (i.e., milk fat content) and perceived advantages for heat and disease tolerance.

**Table 2:** Descriptive of ruminant household livestock holdings (374 households).

	Current total				Prior total (5 years ago)			
	Mean	Median	Minimum	Maximum	Mean	Median	Minimum	Maximum
Overall total (cows/buffalo, all ages)	2.8	2	0	16	3.7	3	0	23
Indigenous cows	0.5	0	0	10	0.7	0	0	15
Crossbred cows	1.3	1	0	7	1.6	1	0	8
Buffalo	0.7	0	0	5	1.1	0	0	15

Among livestock owners, the primary labor sources to maintain livestock was household labor (*n* = 344), with those hiring labor (*n* = 29) having a mean livestock holding of 4.7 livestock, higher than the state mean of 2.80 in our study.

##### Combine harvester use and the fate of combine harvested straw

At the state level (*n* = 374), almost all households (*n* = 360) used some proportion of manual harvesting, 57 used some proportion of the combine harvester, and 6 used some proportion of reaper harvesting (a hand-driven mechanical harvesting device) to harvest rice. Of the households using the combine harvester, the percentage of crop area varied from 20 to 100% with the median use of 60%. Including the purposefully sampled combine users (*n* = 475), of those using the combine below 100% (*n* = 123), the main reasons were to preserve straw for livestock and the inability to access the field with a combine. This is largely supported in our qualitative findings as well.

##### Rice straw as a fodder

Of all 374 households, most households (63.6%) used rice straw from their own fields for feeding purposes. However, it is important to note that there was a significant proportion of households sourcing straw from others.

#### Random forest results

About 32% of the variance in the target variable “burn likelihood” was explained by this model (out-of-bag prediction (OOB) R^2^) with an average 0.16 OOB prediction error (RMSE), i.e., about 16% of the 0 to 1 scale of burning probability. OOB prediction error is an estimate of prediction error in an independent data set. The linear fit (R^2^) of the observed values versus the fitted values (i.e., the fits were predicted using the model created by the same data) was 90.8%). The model overestimated low values and underestimated large values, with a gain greater than one, i.e., a regression of observed on predicted would have a slope greater than one. The reason for the somewhat disappointing performance of the model is likely the need to estimate the burning probability from remote sensing, rather than actual field observations via household surveys. Still, the overall pattern is clear, and we draw some inferences about the importance of each predictive factor.

**Fig. 4:**
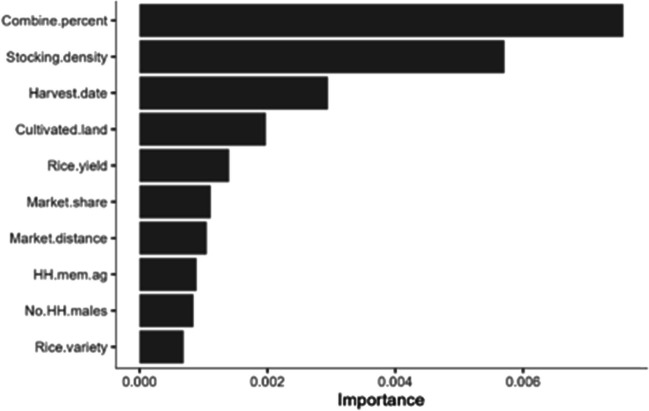
Variable importance plot of random forest model to predict burn likelihood. Units are the increase in mean square error (MSE) if the predictor was randomized

The variable importance plot (Figure 4) shows that the percentage area harvested by combine, livestock stocking density, harvest date, and cultivated land size are the top four predictors of burn likelihood. For combine use, the root mean-square error increases by 0.087 (square root of the MSE 0.0076 as shown in Figure 4) if this factor is randomly permuted. This is 8.7% of the probability, a quite large amount in absolute terms.

**Fig. 5:**
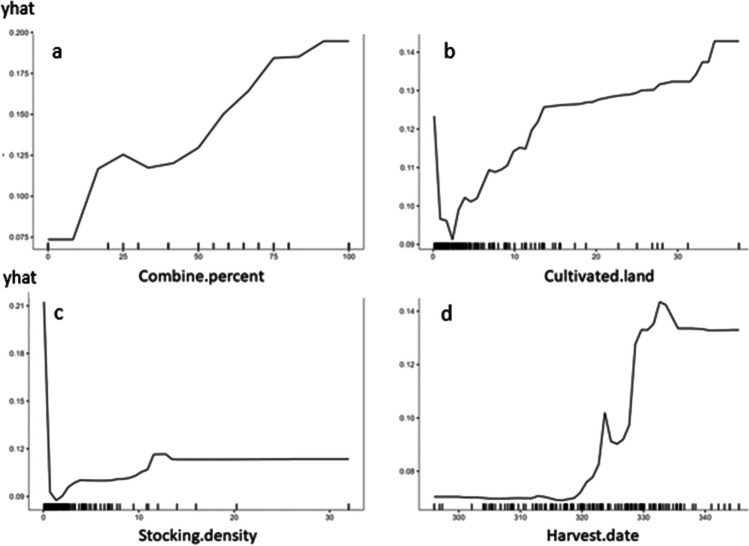
Partial dependency plots of random forest model to predict burn likelihood. Note that “yhat” indicates the predicted burn likelihood based on the values of the predictor variables included in the model

Partial dependency plots were used to describe the relationship between burn likelihood and the four most important predictor variables (Figure 5), holding all other predictors at their median values. As the fraction of combine harvested area on a farm increases, so does the likelihood of burning. Cultivated land area follows a similar trend but may also be serving as a proxy for combine use, as combines more easily service larger fields, as shown in plots of the empirical correlation between predictors and with burn likelihood (Figure S1). There is an odd signal (Figure 5) around very small landholding sizes suggesting that perhaps burning is done because the primary alternative use for the straw is livestock feeding, but yet, these households do not have the physical space to keep livestock and find no value in the straw. The stocking density, or the number of dairy animals divided by cultivated land, shows a strong indication that having zero livestock is highly suggestive of burning practices. With livestock, at any stocking density, the likelihood of burning is much lower. Finally, there is a clear trend with harvest date, with the partial dependency plot showing that a late harvest is more highly associated with burning. This is aligned with other literature (Balwinder-Singh et al. 2019a, b; Ravindra et al. 2019) and anecdotal experiences, noting that later rice harvests result in a shortened, high-pressure planting window which is often addressed by residue burning as a method to quickly clear the field for wheat planting. In Figure 5, we see that stocking density does not necessarily increase with cultivated land increases, which may have implications for livestock systems as future land consolidation occurs, such as has happened in Northwest India.

**Fig. 6:**
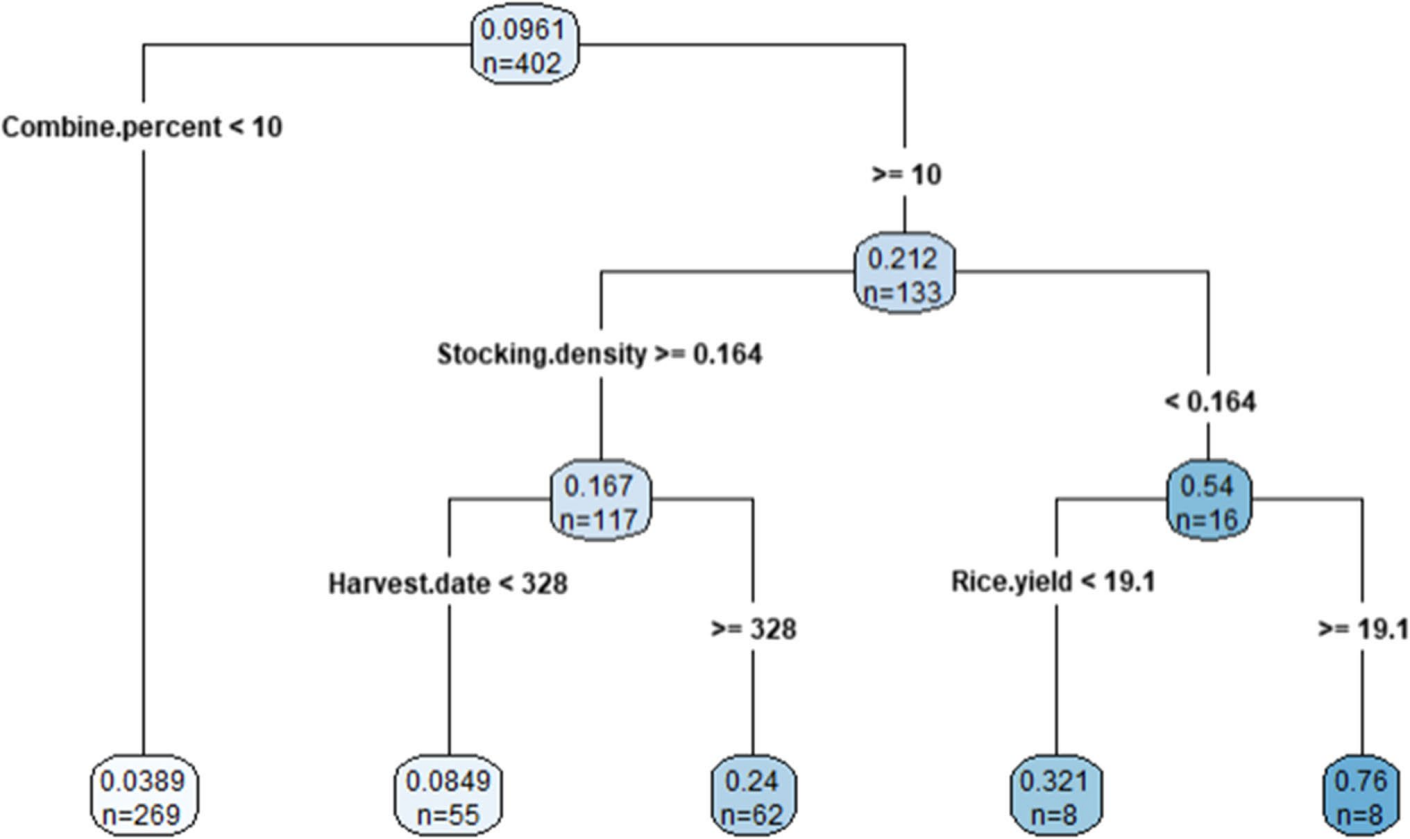
Single regression tree example for residue burning probability to visualize interactions embedded in the random forest model predictions. The values in the nodes indicate the mean of the target variable (i.e., burn likelihood) within the number of observations. For example, in the first node (i.e., root), the mean value of 0.0961 is derived from 402 observations

A random forest has many, mostly sub-optimal, trees. To analyze the tree structure, a simplified, pruned single decision tree was built, using the “rpart” (Therneau 2022) and companion “rpart.plot” R packages (Figure 6). The calculated minimum complexity parameter resulted in a small, three-level tree; so instead, it was pruned according to the visual examination of the complexity parameter table. The combine harvester was the decision variable at the first split, meaning the usage of the combine was the most critical indicator of burn likelihood. It divided the population into two large groups. At the next hierarchical level, for the group with more combine harvesting, livestock stocking density separated these into two groups. Those with lower stocking density were substantially more likely to burn, likely due to less demand for rice straw as a fodder.

A second random forest model was developed using only a subset of combine users (*n* = 133 fully complete surveys) to support a critical policy question: For those who are using the combine harvester, what are important factors that deter burning? With agricultural intensification earmarked to expand in the region, including increased adoption of mechanization, it is important to understand why combine users may not be burning to identify policy entry points. The model explained about 20.5% of the variance (OOB R^2^) with a resulting 0.232 prediction error (OOB RMSE). The variable importance plot (Figure S2) showed the critical relationship between livestock holding, which maintains the value of residue as a fodder, and harvest date, which describes the planting window pressures.

Overall, these random forest models do explain some of the factors related to burn likelihood, especially when combine use is included in the model. However, there are more intrinsically complicated drivers of burning related to social, perceptional, and political actors that are not represented in our predictor variables. Arguably, these quantitative methods do not adequately capture the inter-variable dependency, nor the order of system changes, that enable us to adequately characterize the system. Therefore, these aspects are further explored through the qualitative aspects of this study. Additionally, our burn likelihood target variable may be a somewhat unreliable measure of actual household burning activities. First, this response variable is from the kernel density estimation of satellite-derived fire observations, not actual burning reports or ground observations of the households. Similarly, the use of the satellite-derived fire estimates misses the majority of small fire activity, due to time of overpass, cloud cover, and haze. However, this exercise does give us important insights into the drivers of burning.

### Qualitative results: drivers of cropping system changes

**Fig. 7:**
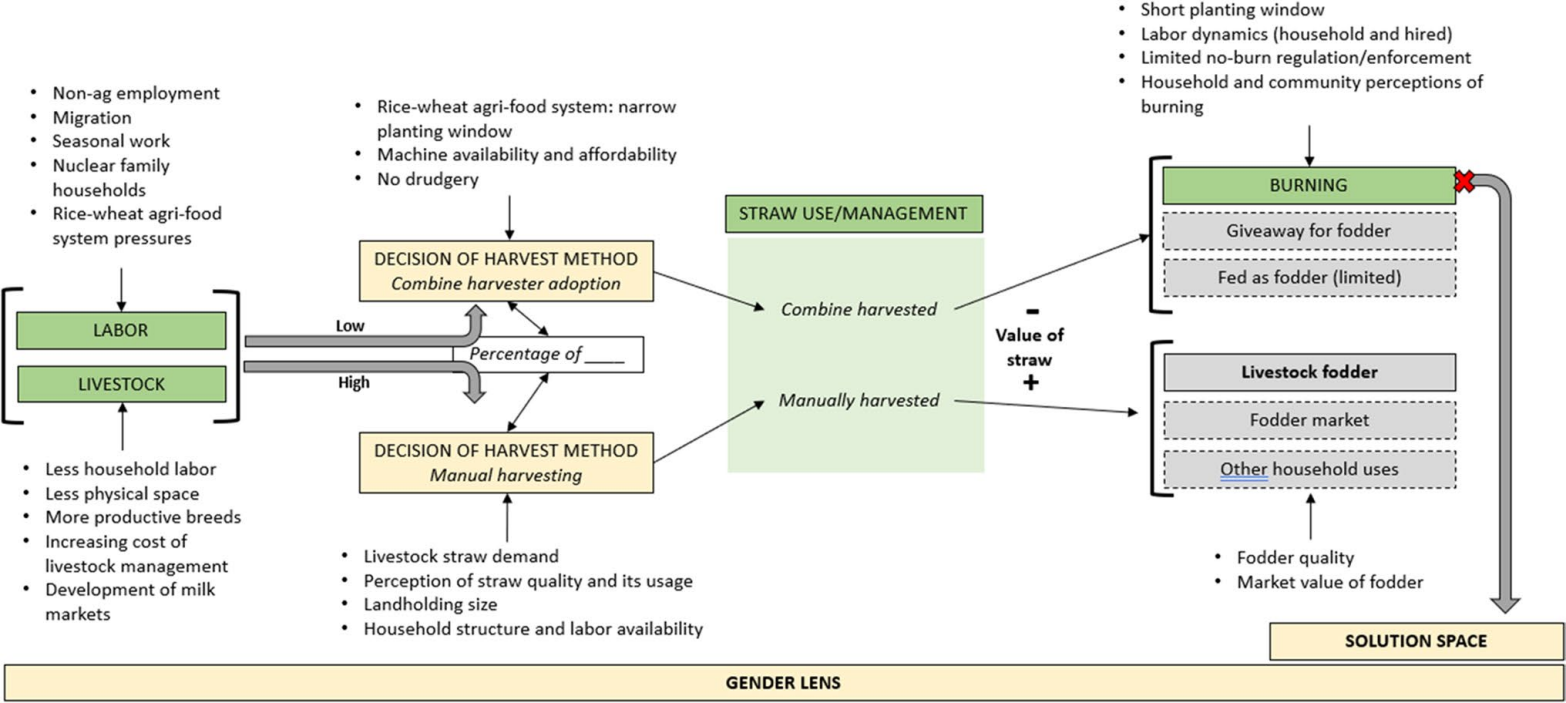
Conceptual model of factors influencing rice harvest and residue management

The LPA framework was primarily used during qualitative analysis and codebook development to ensure all resources (i.e., human, financial, physical, knowledge) and structural levels (i.e., individual, household, community, institutional) were considered. This evaluation, guided by the LPA framework, identified several factors influencing rice harvest and burning and described how these factors cascade in a sequence of events, particularly at the household level. Figure 7 provides a conceptual model of factors influencing rice harvest and residue management. Factors in green are central factors (Figure 7).

Thirty-six respondents, 18 male and 18 female, from 18 households, participated in the qualitative component of this study in the villages of Lalachak, Chotaki Basauli, and Rajapur of Buxar district. All households primarily followed a rice-wheat cropping system, with cultivated land size ranging from 0.4 to 4.9 ha (mean = 2.1 ha). Household herd size ranged from one to six adult dairy animals, with household labor ranging from one to thirteen people (mean = 5). Half of the households had burned rice residue at some point in history. In the subsequent quotes, respondents are labeled with an “F” or “M” to signify either female or male so the reader can capture gender nuances in the quotations. For example, “4_M” is the fourth respondent who identifies as a male.

#### Main drivers of harvest method decision: labor and livestock

The decision of harvesting method, including the fractional use of the combine harvester versus manual harvesting on total cultivated area, is central to the ultimate fate of residue in this cropping ecology. If livestock holding is high at the household level (physical resource), household demand for fodder is high and the household is more likely to use a greater percentage of manual harvesting to meet the fodder demand. Manual harvesting preserves straw quality by maintaining the long, straight, clean stems of the crop. Multiple respondents stressed that the percentage of farmland that is manually harvested is determined according to animal feeding needs, including:

Now only the amount [of straw] to be used in animal feeding is cut by hand and the rest of it is cut by harvester. (17_M)

[Manual harvesting] is done according to the requirement of animal food. (21_M)

While livestock fodder needs are central to the continued practice of manual harvesting, there is strong evidence that livestock numbers are decreasing at the household level. Our interviews suggest that trends in livestock holdings are largely associated with the decrease in available household labor (human resource). Respondents explained:

Now almost all families are the nuclear family [compared to large extended families] so they have less members than before and everybody is busy earning money. That is why they don’t have sufficient time to look after the animals. (5_M)

Now, [the number of animals kept] has reduced as there are less members in the family. And everyone is busy in their work and they go outside and don’t have time to take care of the animals. (7_M)

On multiple occasions, farmers mentioned the increase of milk productivity of improved cattle breeds (physical resource), generally in the context of crossbreeds, and therefore did not need to keep as many livestock compared to the past. However, these breeds require high levels of maintenance (human resource) and require changes to feeding strategies (physical resource). Another household trend is the positive views of buffalo due to the higher milk fat content over cattle and heat tolerance. This quote gives insights to milk production changes with improved breeds and feeding strategies, “Earlier [cattle] used to give 1-2 liters of milk but now jersey cows have come. If you give them good quality fodder, then they can give you up to 10 liters of milk” (15_M).

Labor, both at the household and community levels, is a critical driver of the rice harvesting decision. Manual harvesting is highly labor and time intensive in the time-sensitive rice-wheat cropping season. Farmers are acutely aware that timely planting of wheat is directly associated with higher wheat yields. Respondent 9_M explained, “The [wheat field] which is sown first will grow a good crop and the one which is sown later will not have a good crop.”

With household labor (human resources) availability trending downward due to off-farm employment, studies, and migration, the need to hire labor to manually harvest rises. Yet, the availability and cost (financial resources) of hired labor are often not worth the comparative benefit, as respondent 11_M explained, “Now there is a scarcity of [laborers] so people think that they should harvest it by hand only for their cattle.”

#### Harvest method drives perceived straw value and fate

The perceived value of straw is directly related to the way in which the rice crop was harvested. In this ecology, the main use of rice straw is for feeding dairy cattle and buffalo. Farmers use both rice and wheat straw for feeding based on the harvest and season. Combine harvested straw deviates from the preference for straight, clean stalks that are made into bundles in exchange for those which are short, loosely scattered in the field, crumpled, and sometimes soiled and with higher moisture content making it difficult to collect and make a proper straw bundle. For example, “Hand cut straw can be stored for a long time but machine cut straw spoils quickly,” explained respondent 17_M. Combined harvested straw was viewed as a waste product this is primarily disposed of through burning. While manually harvested straw holds value as a fodder to feed cattle, combine harvested straw is largely rendered “useless.”

When mentioned, all respondents noted the superior quality of manually harvested straw over combined harvested straw; however, their reasoning varied. Some of the respondents fed combine harvested straw nonetheless. The following quotes provide insight to these perceptions:

… for cows, the hand cut straw produces better quality milk. It is thicker and tastier than the machine cut straw in which the milk is less tasty and less thick. (8_F)

Straw harvested by hand will be good for [the livestock] whereas straw harvested by machine are just like dust. (9_M)

There was evidence of fodder markets for manually harvested rice straw, with reported prices ranging from 1000 to 2000 rupees ($12.21–$24.42 USD; Exchange rate given as 81.9 INR/USD (April 2023)) per 100 kg with a seasonal variation, as the price is lower immediately after rice harvest and goes up before wheat harvest. However, there was no evidence of households hiring labor to hand harvest for the sole purpose of marketing, inferring that market prices (institutional level; financial resource) are not high enough to incentivize manual harvesting on its own. Manual labor was reported to cost 250–400+ rupees ($3.05–$4.88+ USD) per day plus two meals. Fodder demand trends may diverge in different in other regions of Bihar, such as in areas south of Patna where large milk cooperatives exist.

There were a variety of responses related to female involvement in the decision of harvesting method, as well as crop and livestock involvement (Text S3).

#### Burning

In this region, crop residue burning is a very sensitive topic because of the fear of social stigma and the risk of punitive actions. Hence, asking informants directly about their own burning practices can lead to untruthful responses. For example, one respondent explained, “… about 60-70% people are burning the straw” (19_M), yet in the same village, another respondent expressed, “Not one of us burns the straw in this village” (21_M). Therefore, we approached the interview topic from an angle that avoided individual household burning activities and focused on understanding burning drivers and perceptions at the community level.

The physical reasons expressed for rice residue burning at the community level were related to field preparation for wheat. These respondents explained the reasons for burning:

If anyone is farming on a lot of land, then [burning] is the easiest way to clean the land as there is a shortage of labor and it will be expensive and time consuming. Also, there is a reduction in home animals now so there is no such use of the straws. (8_F)

[Burning is done] to get the next crops planted at the right time. There is a problem of labor so by burning the straws, the land becomes clean very fast and also the pests are killed and because of this, the production increases. (7_M)

[Burning is] for timely sowing and reaping, for lack of labor, for saving time and money and to increase the fertility power of the land. (31_M)

The most common reason for not burning was to preserve the straw for livestock. When there is a household demand for fodder, the rice fields are manually harvested to maintain the quality of the straw as fodder. Respondent 33_M explained, “Approximately 90% [of the village] has burned. Whomever has animals in their homes uses [the rice fodder] as their feed, otherwise, those who don’t have animals to feed burn it… More livestock, less likely to burn.” Another respondent explained, “Not one of us burns the straw in this village… Because they store [the manually harvested straw] to sell it and use it to feed the animals” (21_M). Uniquely, one respondent noted the use of the straw as fertilizer. “In our village, 30% percent burn the crops and the other 70% store [the residue] for one year and use it as fertilizer” (29_M), although many respondents mentioned that synthetic fertilizers have made this practice obsolete.

Our coding strategy identified four main perception categories related to burning, including the impact of burning on the cropping system and soil fertility, the impact on air quality and human health (Text S4), the governmental rules, regulations, and enforcement related to prohibiting burning, and perceptions of alternative no-burn options.

When asked about their perceptions of government regulations and enforcement against burning, respondents’ responses varied among being very aware of the legal risks of burning, to being aware but not concerned, to not being aware of any no-burn regulations, including:

No, there are no restrictions, and no one came [to our farm] to see, so we have no fear of anyone (i.e. the government officials). (33_M)

[People in the community] do [burn] but with great concern. They have fear of police and administration. (11_M)

I don’t have any fear of anyone. It is my land so I’m burning the straw on my land. (7_M)

[Government officers] came here but nobody is ready to stop this [burning] process. (19_M)

Respondent 19_M later explained that farmers do have some fear of the government so opt to burn at night to avoid being seen. Others mentioned this as well, but the majority reported burning or seeing others burn in the afternoons. As this respondent and others mentioned, enforcement of no-burn regulations is limited. When asked why, it was explained, “Because [the enforcers] also do the same [burning]. They also have to do farming” (13_M). Additionally, a type of “community action” takes place to avoid being punished and to stand against enforcement by waiting to all burn at once or to support fellow farmers when confronted by enforcers. One farmer explained, “When people see that someone is burning their field, then they also burn their own” (11_M). Another farmer explained that there is little government pressure during rice harvest, but in the context of the wheat harvest, there are some restrictions and noted that “When everyone is ready to burn only then we have to burn it” (3_M).

Overall, farmers are aware of the negative implications of burning and the increasing government pressure against it. Towards the conclusion of each farmer interview, we probed respondents for suggested solutions to halt burning. Overall, they found this challenging, with many responding that there was no other option (Text S5).

## Discussion

Farmer decision-making was evaluated using the Livelihood Platforms Approach, which not only recognizes specific resource availability, namely, physical, financial, human, and informational but also recognizes the hierarchical structure in which farmers and the available resources are situated in, namely, individual, household, community, and institutional levels. Mixed methods were utilized in this study to move beyond a single disciplinary perspective. While this approach was taken in response to the needs identified in the larger body of international literature (Malawska and Topping 2016; Singh et al. 2016), the most critical finding was the strong presence of a sequential decision-making process in these landscapes (Robert et al. 2016). This study suggests that there is a clear sequencing of events, primarily at the household level, that lead to an increase of rice residue burning in Bihar. As such, decision processes should be considered across space and time (Robert et al. 2016). Characterization of this cascading sequence can inform targeted policy interventions to achieve no-burn goals. Osman (2010) acknowledges the human capacity to find control within complex, dynamic environments, with problem-solving activities assuming a central role. We argue that adopting a broad lens encompassing spatio-temporal change processes in landscapes can not only enhance researchers’ comprehension of farmer decision-making but also inform problem-solving decisions and efforts pertaining to agroecological challenges.

The random forest analysis, along with the qualitative interviews, showed a positive relationship between the probability of burning and combine harvester use, similar to research results from Northwest India (Kumar et al. 2015). Recognizing that farmers burn straw for quick disposal to facilitate wheat sowing, this study showed that burning is more prevalent with late rice harvesting, with farmers acutely aware, qualitatively, that late wheat planting negatively impacts yields and thus profitability. The question is whether technological interventions and policy efforts across the Indo-Gangetic Plain have integrated sufficient safeguards to protect the interest of farmers. In this Bihar-focused study, harvest method (i.e., combine harvester versus manual harvesting) was strongly mediated by fodder demands that are determined by bovine herd size at the household level. This study does not address state-wide livestock trends, among our surveyed households, herd size is decreasing, and the primary reported reason for this decrease is reduced household labor availability. With local labor markets anticipated to continue to tighten in Bihar (Imbert and Papp 2020; Nayyar and Kim 2018), it is important to recognize how structural changes in the rural economy may impact ecosystems services and public health in the absence of mitigation measures (Urban Cordeiro et al. 2023).

Household labor availability appears to be the first step in the series of cascading changes. As it relates to livestock feeding and tending, it is critical to acknowledge *who* in the household makes decisions and *who* carries out day-to-day tasks around livestock, so that programming can be targeted appropriately. The vast majority of households interviewed relied on household labor for livestock tending, rather than hired labor. Despite commonly understood perceptions of gender roles in this region (Ravichandran et al. 2021; Sinha 2007), our findings suggest that men spend more than double the time than women carrying out daily livestock tasks. Our qualitative analysis suggests that many women respondents were not involved with livestock, even disinterested, with the male head of household, and sometimes children, taking main responsibility. From a cropping system perspective, women respondents were largely uninformed and uninvolved in cropping system activities, which was the assumption given the dominantly patriarchal context of this district, as described by local gender experts. Yet, there were some women who stated that they jointly made decisions with their husbands, including decisions around combine use and burning. We recognize that the functional definition of joint decision-making within these households varies (Acosta et al. 2020), but these findings suggest that the involvement of both men and women need to be considered when designing sustainable development interventions (Zegenhagen et al. 2019).

In Eastern India, livestock feeding imparts value to rice straw, similar to what has been found in the Terai region of Nepal (Bajracharya et al. 2021). The state of Bihar has acknowledged dairy investments to achieve other poverty, dietary, and social equity goals (Bihar Livestock Master Plan 2018), but explicit linkages to burning are absent in current policy frameworks. Creative policy approaches could support crop-livestock integration at the household and village levels. More specifically, small to medium-scale commercial models for dairies that depend on local residue sources and overcome household labor bottlenecks for maintaining livestock could provide a model of sector development that aligns with prevailing demographic trends. Evidence in this study suggests that milk markets are formalizing and farmers are adjusting livestock type and feeding practices to secure the higher payments for higher quality milk (i.e., high milk fat content). Incentives need to maintain local fodder demand as milk markets formalize, otherwise there is a risk of creating sub-regional patterns of fodder surplus and deficits that could lead to accelerated transitions to burning in residue surplus areas. Development programs that conserve crop-livestock system integration will help ensure that local fodder demand and supply are roughly in balance and that the value of residues is maintained. Since residue markets are dominantly local given the high cost of transport for a low value commodity, the role of market facilitation for residues appears to be limited.

Policy interventions targeting local labor and livestock considerations should be prioritized given our findings of cascading drivers of system change, but a package of technical and financial levers could accompany these efforts. *In situ* straw management through technologies like the Happy Seeder may have a role in the long-term, but given the limited adoption among comparatively well-resourced farmers in Northwestern India, scope for similar technologies in Eastern India are likely very limited in the near-term (Shyamsundar et al. 2019). Unlike Northwest India, there are landscape and environmental factors that will limit full adoption of the combine in Bihar, such as small plot sizes and seasonal field wetness that limit the operating window for the combine (Shambhu and Jha 2012). As such, there is an opportunity to promote other labor saving technologies, such as reapers, which have seen some success in similar landscapes in Nepal (Brown et al. 2021). Reapers are both optimized for preservation of residue value and can be readily used within the patchwork of smallholder fields that characterizes Eastern India. Also, evaluation of companion technologies for the combine, such as straw bailers, may facilitate ease of collection and storage while maintaining quality (Manjunatha et al. 2015).

Other functional levers include reducing pressures on the planting window for wheat, such as through the promotion of direct-seeded rice and shorter duration rice varieties (Balwinder-Singh et al. 2019a, b). Finally, the burgeoning carbon finance markets for soil carbon sequestration and greenhouse gas emissions reductions could provide a potential opportunity to financially incentivize farmers to maintain the residue in their systems. Through both functional and financial levers, Bihar policy makers can incentivize system change in a sustainable direction. No-burn regulations are currently in place; however, from this study, farmers who burned were quick to defend the practice as the only means to clear the field for wheat and maintain their farming livelihoods. Left with no choice, and despite government no-burn policies, there was no indication that burning practices were likely to reduce on their own. As such, a package of technical and financial levers needs to accompany larger policy efforts targeting local labor and milk markets.

## Conclusion

Rice residue burning is increasing in Eastern India. This mixed methods study characterizes the drivers contributing to burning transitions in the state of Bihar through the lens of socio-technical change.

Factors promoting burning are primarily taking place at the household level, with influences at community and institutional levels through the introduction of the combine harvester through the mechanized service economy. Changes at the household level occur as a cascading sequence of events, starting with decreases in household labor leading to decreases in household herd size and demands for livestock dry fodder in the form of crop residues. Where the combine harvester is available, residue demands dictate choice of harvest methods with combine harvesting strongly associated with rice residue burning. Novel policy approaches are needed to recognize these dynamics. For example, policy efforts targeted at commercializing local milk markets may foster crop-livestock integration and discourage burning. Additionally, the expanding carbon finance opportunities related to soil carbon sequestration and the reduction of greenhouse gas emissions offer a potential avenue for financially motivating farmers to retain residues. Further research should expand the qualitative work of this study beyond a single district in Southwest Bihar to capture the heterogeneity of the state. Interviews with key informants involved in milk and straw markets at the district and state levels would add an additional depth of understanding to community and institutional level drivers of change. Before the practice of burning becomes “locked in,” Bihar state has a narrow window of opportunity to test the efficacy of alternative policy approaches even as broader demographic and technological changes accelerate.

Our study contributes to the broader agronomy for sustainable development literature by exemplifying how a comprehensive system understanding can be achieved through the integration of mixed methods and the collaboration of multidisciplinary experts, including system agronomists, social scientists, and data scientists. Guided by a theoretical framework emphasizing hierarchical levels of interactions (i.e., household, community, and institution), our research not only identifies drivers of change but the order and societal level in which they occur. This approach yields valuable insights for global research-for-development practitioners, aiding in the development of policies that are proactive to pending sustainability crises.

## Supplementary Information

Below is the link to the electronic supplementary material.

Supplementary file1 (DOCX 91 KB)

## Data Availability

Data from this study are available from the corresponding author upon reasonable request.

## Code availability

The code generated for analysis in this study is available from the corresponding author upon reasonable request.
