# Supplementary material for: Transitions to crop residue burning have multiple antecedents in Eastern India
Source: Agron Sustain Dev. 2024 Nov 7;44(6):59. doi: 10.1007/s13593-024-00983-3 (PMC11543722; doi:10.1007/s13593-024-00983-3)
Supplement: Supplementary file 1 — Supplementary file1 (DOCX 91 KB) [file 13593_2024_983_MOESM1_ESM.docx]

**Supplementary Information for**

**Transitions to crop residue burning have multiple antecedents in Eastern India**

| **Table of Contents: Supplementary Information** | | |
| --- | --- | --- |
| Text S1 | Results: Descriptive statistics (cont.) | |
| Figure S1 | Correlation plot and histograms of key variables of interest | |
| Figure S2 | Variable importance plot of random forest model to predict burn likelihood for combine harvester users only | |
| Text S2 | Main drivers of harvest method decision: Labor and livestock (cont.) | |
| Text S3 | A gender lens on straw value and fate | |
| Text S4 | Perceived impacts of burning on the cropping system and air quality | |
| Text S5 | Respondents’ suggestions to halt burning | |
|  | |  |

**Text S1. Results: Descriptive statistics (cont.)**

**Livestock (cont.):** When asked how many hours per day it takes to carry out all livestock-related activities by adults (18 years+), respondents reported an average of 3.7 male hours and 1.6 female hours, suggesting men have a greater daily involvement with livestock than women. This finding was supported by qualitative interviews and does counter the prevailing notion that women are the primary caregivers for livestock in the region. Related to milk market integration, 197 (55.3%) households selling their milk and 159 (44.7%) households not selling their milk, with the primary sales locations being neighbor(s) (*n*= 96; 48.7%) and the local co-op (*n*= 83; 42.1%).

**Combine harvester use and the fate of combine harvested straw (cont.):** Of those using a combine, most began around 2016, reporting that the combine is an easier and faster method for harvesting. However, the primary reported challenge with the combine is its limitation for small plot sizes. Other challenges included some lack of availability during key harvest times, a decreased value or usability of straw, and the additional effort needed to manage straw on the field. Our qualitative interviews strongly suggested that timely combine availability was not a barrier to use in Buxar district; in fact, respondents noted that it was easier to find a combine to hire than laborers to manually harvest. 68.6% of combine users anticipate that they will hire a combine to harvest more of their rice area in the next five years.

All households (*n*= 475) were included to examine straw fate of the harvesting methods to ensure substantial combine user respondents (*n*= 156). After the combine harvester, straw (multi-select) was collected by hand (*n*= 147), left in field and was used for grazing (*n*= 115), incorporated into the soil (*n*= 32), left on surface (*n*= 16), burned (*n*= 6; full stubble only), and/or collected by a bailer (*n*= 3). After the harvester, the following machines (multi-select) were used to prepare for and plant wheat or the next crop: cultivator (*n*= 87), rotavator (*n*= 68), and/or zero-till drill (*n*= 62).

**Rice straw as a fodder (cont.):** Of those who sourced straw (purchased and/or free) (*n*= 120), 50% sourced more than half of their required straw from others. Rice straw sellers (*n*= 120) sold manually harvested straw, with the majority of buyers categorized as other farmers in the same village (*n*= 94) rather than traders (*n*= 26), implying that most straw markets are highly localized. Of those who sold manually harvested straw, 56.7% sold more than half of their total straw. Of the 120 farmers who sold straw, 113 reported that they could have sold more manually harvested straw if they had more available. That said, we found no evidence in the qualitative interviews suggesting farmers sometimes chose to manually harvest over combine harvesting for the sole purpose of selling more high-quality straw.

To understand if combined harvested rice straw has a market value, we summarized the responses from all surveys, including the purposefully selected combine users (*n*= 475). Among the 156 households who used a percentage of the combine for rice harvesting, 19 total sold the combine harvested straw in the form of chopped (*n*= 6), loosely collected (*n*= 9) or in a straw bundle (*n*= 4). The buyers were roughly split between farmers and traders and roughly split in location between the same village and neighboring villages. These totals suggest that combined straw has a very limited market.


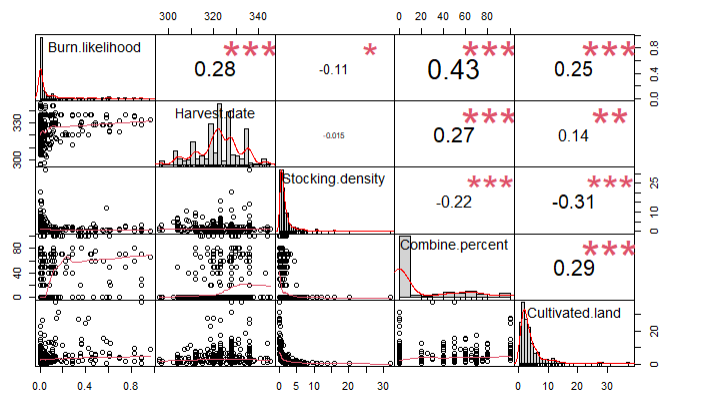


**Figure S1:** Correlation plot and histograms of key variables of interest. The variables are displayed as histograms (center diagonal). Scatter plots display the relationship between the predictor variables (bottom left corner), with the correlation coefficient presented (top right corner).


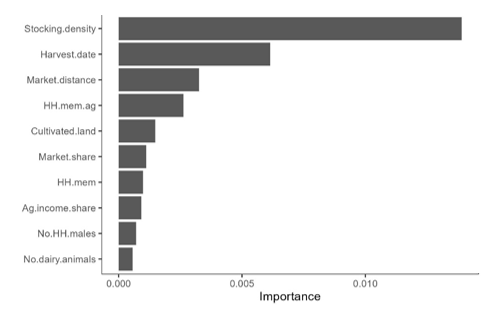


**Figure S2.** Variable importance plot of random forest model to predict burn likelihood for combine harvester users only. Units are the increase in mean square error (MSE) if the predictor were randomized.

**Text S2.** Main drivers of harvest method decision: Labor and livestock (cont.)

This respondent described:

“No laborers are available to harvest [the rice]. They are all going to other cities. They do worse work than [harvesting], but they don’t work here… Today is a modern world and people don’t work like they did before. Earlier people would go a very far distance carrying a sickle in their hand… Now, the generation has changed.” (13_M)

This also provides insight into how the community perceives the value and status of agricultural labor. There is a trend to move away from agricultural labor work. At the same time, some have the perception that there is also a ‘pull’ out of agriculture by institutional level drivers, such as subsidy programs as suggested by one respondent noting:

“[The laborers’] stomachs are getting full by going to the cities. They are enjoying different schemes available by the government. If the harvester was not present here, we would have died of starvation. [Farmers] are all [harvesting rice] by harvester.” (15_M)

**Text S3. A gender lens on straw value and fate**

The decision of harvesting method is primarily determined by the male head of household, with often women knowing little to nothing about the cropping activities of the household. Some examples include:

“Actually, I don’t know about [the harvesting decision] because I stay at home and only males look after this matter.” (4_F)

“We can’t go outside our door.” (16_F)

However, there were multiple contrasting examples that gave insight into much more female involvement in cropping, including working in the field and joint decision making with their husbands. Example statements include:

“I and my husband decide jointly.” (30_F)

“My husband and I jointly take decision about [the harvesting method]… My husband does everything [related to farming] but I help him sometimes.” (34_F)

**Text S4. Perceived impacts of burning on the cropping system and air quality**

The responses from the perceived impacts of burning on the cropping system, and soil fertility specifically, were viewed both negatively and positively. These respondents explained the connection, or lack of connection, between burning and fertility:

“After burning the residue, the field gets strength. It provides fertilizers.” (15_M)

“Production will be better in both [burned and not burning fields] because production directly depends on [commercial] fertilizers today.” (10_F)

Yet, this response from respondent 23_M demonstrated a mixed perception of the issue: “I think there is both loss and gain [associated with burning]. The loss is that the land becomes dry and soil moisture reduces, and the gain is that it is time saving as it helps to reap and sow fast and also reduces the labor charges.” There were multiple mentions of the connection between burning and pollution, as farmers seemed to be aware of this negative implication of burning. Multiple respondents echoed a similar statement to this, “There are some disadvantages to burning… the smoke emitted from burning spreads air pollution and can cause dangerous effects on the lungs and respiratory system” (19_M).

**Text S5. Respondents’ suggestions to halt burning**

Respondents stated:

“[The government] publishes [the names of farmers caught burning] in the news … But what will we do if we do not burn it? …If we do not burn it then how do we sow other crops? We know that it is very harmful to burn. …There is no benefit by burning it, yet we have to do this.” (11_M)

“[Burning] is absolutely wrong… I have created [the crop] and I am destroying it, but it is my helplessness. So I can’t do anything because there is no other option… We know that it is very harmful to burn… There is no benefit by burning it, yet we have to do this.” (11_M)

However, some respondents did offer more specific details in the solution space, which includes the use of rice straw as fodder, rice straw as fertilizer, monetary incentives to farmers, development of machines for straw management. Example statements included:

“If people start to keep more animals again at home, then the straw is used as fodder for the animals and it could be stopped. And also, if the government imposes a law against burning, it will also be helpful.” (19_M)

“I think the only solution is for companies to make fertilizers of the machine cut straw.” (21_M)

“Government should impose a penalty system on it by taking strict actions.” (5_M)

“I think the government should take initiative regarding this. If the government offers some monetary help to farmers to scale farming, then I think the farmers would also listen to the government to not burn the straw.” (29_M)
